# A comparison of the effectiveness of three LED phototherapy machines, single- and double-sided, for treating neonatal jaundice in a low resource setting

**DOI:** 10.1371/journal.pone.0205432

**Published:** 2018-10-11

**Authors:** Gaston Arnolda, Tran Dinh Chien, Andrew Hayen, Nguyen Thi Xuan Hoi, Katherine Maningas, Priscilla Joe, Francesco Cavallin, Daniele Trevisanuto, Luciano Moccia

**Affiliations:** 1 School of Public Health & Community Medicine, Faculty of Medicine, University of New South Wales, NSW, Australia; 2 Thrive Networks, Oakland, CA, United States of America; 3 School of Public Health, Faculty of Health, University of Technology Sydney, NSW, Australia; 4 Division of Neonatology, Children’s Hospital and Research Center, Oakland, CA, United States of America; 5 Independent statistician, Solagna, Italy; 6 Amici della Neonatologia Trentina, Trento, Italy; 7 Department of Woman and Child Health, Medical School, University of Padova, Padova, Italy; 8 Day One Health, Redding, California, United States of America; Massachusetts General Hospital, UNITED STATES

## Abstract

**Introduction:**

Neonatal jaundice is one of the most common reasons for hospital admission in low resource settings. Treatment is frequently inadequate as conventional phototherapy requires frequent bulb changes. LED phototherapy has comparable efficacy to conventional phototherapy, and the bulbs last over 40,000 hours. This observational study compares the effectiveness of three LED machines, two single-sided and one double-sided in routine use in Vietnam.

**Methods:**

We included all infants weighting ≥1500g and with jaundice diagnosed visually or by Total Serum Bilirubin (TSB) measurement at The Da Nang Hospital for Women and Children (Da Nang, Vietnam). The primary endpoint was the average hourly change in TSB over the first six hours of treatment. The secondary endpoints were duration of treatment; average hourly change in TSB over treatment, and length of stay in the neonatal unit. Multivariable analysis and bootstrap methods was performed to compare outcomes, adjusting for potential confounders.

**Results:**

All outcomes were comparable in the two single-sided machines. The double-sided machine showed 54% increase in the hourly speed of TSB reduction (1.3 μmol/L/hr, 95% CI 0.3–2.3), with a 45% increase in the speed of TSB reduction over the duration of treatment (0.9 μmol/L/hr, 95% CI 0.6–1.3). In addition, the double-sided machine was associated with 21% reduction in the duration of treatment (14 hours, 95% CI 5–22) and 16% reduction of length of stay (14 hours, 95% CI 3–25).

**Conclusion:**

The results confirm and quantify the benefits of increasing surface-area exposure during phototherapy. Adjusted for multiple potential confounders, use of double-sided phototherapy can substantially increase the speed of TSB reduction, and substantially decrease the duration of treatment and length of stay in the NCU.

## Introduction

Neonatal jaundice is a common condition affecting over 60% of neonates, and frequently requiring medical attention [1]. While usually benign, sustained elevated bilirubin levels can occasionally result in permanent neurological damage or death, but these outcomes can be prevented through appropriate management. This has been largely, but not entirely, achieved in high-resource settings [2, 3].

In low resource settings (LRSs), treatment of neonatal jaundice is often sub-optimal [4, 5]. Conventional fluorescent light phototherapy is often the only available option in LRSs, and the irradiance provided is usually below recommended levels [6–8]. Common reasons for low irradiance include: using white fluorescent bulbs with low irradiance in the efficacious spectrum for treatment of jaundice; and failure to replace blue bulbs on a regular basis (typically 1,000–2,000 hours) due to cost, lack of a photo radiometer to monitor irradiance, or lack of an on-machine counter to track hours of use. Sub-optimal phototherapy treatment can lead to long lengths of stay while jaundice resolves spontaneously, and the lack of effective phototherapy can lead to a need for exchange transfusion [8]; both outcomes pose additional risks to neonates, and have resource implications for facilities caring for sick neonates in LRSs.

A Cochrane Review found that LED and non-LED (halogen or fluorescent bulb) phototherapy result in a comparable rate of reduction of total serum bilirubin (TSB) [9]. The cost of LED phototherapy has reduced markedly in recent years, and LED phototherapy bulbs typically have a 40,000–50,000 hour lifespan, eliminating the need for frequent replacement of bulbs, making it an affordable technology for LRSs and creating the opportunity for high quality phototherapy to be consistently provided in LRS.

The aim of the present study was to compare three different LED machines, two single-sided and one double-sided, in speed of reduction of TSB, duration of treatment with phototherapy, and total length of stay (LOS). The study was undertaken as part of a program of operational research to inform programming decisions by an international non-government organization (INGO) that donates LED phototherapy to hospitals in LRSs.

## Materials and methods

### Setting

The Da Nang Hospital for Women and Children, located in south-central Viet Nam, had 13,411 births in 2014 and its Neonatal Care Unit (NCU) accepted 3,934 admissions, born at the hospital or transferred in, of which 1,207 (30.7%) were for jaundice. The hospital had several LED and conventional phototherapy machines prior to study commencement. Neonates were enrolled in the study from May 2013 to September 2014.

### Study population

The study population was restricted to infants born weighing ≥1500g, with jaundice diagnosed visually or by TSB, and having their first episode of phototherapy at the hospital. Infants with gestational age <33 weeks, those with axillary temperature <36.5°C or >37.5°C at treatment commencement and those treated in an incubator were excluded.

### Intervention

The study compared three LED phototherapy machines specifically designed for LRSs (Table 1). Two machines provide single-sided phototherapy from above (“PTV3000” and “Lullaby”), while the third (“Firefly”) is double-sided. The lamp heads of both single-sided machines can be adjusted to provide therapy at various distances from the baby, altering the irradiance, while the double-sided device provides treatment at a fixed distance from both above and below. The Firefly has a single irradiance setting while both single-sided machines have two settings and can be put into high-irradiance mode by flicking a switch; the PTV3000 automatically reverts to standard mode after 3 hours, while the Lullaby must be manually switched back. No restrictions were placed on the hospital’s use of the single-sided machines, in relation to the distance of the lights from the neonate, or the irradiance setting to be used.

**Table 1:** Key features of the three LED phototherapy machines.

	PTV3000	Lullaby	Firefly
Manufacturer	MTTS, Hanoi, Vietnam	GE Healthcare, Maryland, USA	MTTS, Hanoi, Vietnam
Type	Single-sided	Single-sided	Double-sided
Weight	18.4 kg	10 kg	13 kg
Irradiance settings	Two: Standard/High [High setting reverts to low automatically after 3 hours use]	Two: Standard/High [High setting is changed by manually flicking a switch]	Single setting
Fixed/adjustable treatment distance	Adjustable	Adjustable (and head is detachable allowing place atop an incubator)	Fixed
Irradiance from above*	Standard: 25 μW/nm/cm^2^ High: 40 μW/nm/cm^2^ [Measured at 40 cm]	Standard: 22 μW/nm/cm^2^ High: 45 μW/nm/cm^2^ [Measured at 35 cm]	32 μW/nm/cm^2^ [Fixed distance]
Irradiance from below*	N/A	N/A	36 μW/nm/cm^2^ [Fixed distance]
LED bulb type [dominant wavelength]	Luxeon Rebel Royal Blue, High Power [450–475 nm]	Cree [450–465 nm]	Seoul Semiconductor Blue High Power [453–465 nm]
Number of bulbs	42	10	10 above + 18 below
Expected bulb life^#^	50,000	50,000	44,000

* As per manufacturer’s specification.

^#^ Estimated hours of use before reaching 70% lumen maintenance (i.e., 30% reduction in irradiance)

### Allocation

While the study was observational, an un-blinded system of quasi-randomisation was devised. As the NCU had limited space, only one machine of each type was provided for the study, and it would have prolonged the study if infants could only be allocated when all three machines were available. Eligible infants were allocated in a standard order, based on a number assigned to each device: the PTV3000 was assigned #1, the Lullaby #2 and the Firefly #3. Allocation was to the available machine where only one was available, or to the lower numbered machine if two or more were available.

### Data

To minimise the data collection burden, no data was collected on eligible babies that were excluded for any reason. For enrolled infants, data was collected on several factors including: estimated gestational age; birthweight, date and time of birth; inborn/outborn status; sex; maternal and infant blood type and direct Coomb’s test; Rh(D) status; G6PD deficiency; previous sibling received phototherapy; significant bruising; exclusive breastfeeding and feeding poorly; date and time and TSB (μmol/L) at treatment start and at end, and TSB at 6 hours; date and time of nursery admission and discharge; selected treatments that could influence length of stay (oxygen; antibiotics); machine allocation; and machine used for treatment. Data was collected by hospital clinicians on individual paper forms, and forwarded to project staff for entry in a database.

### Endpoints

The primary endpoint was change in TSB/hour, over the first six hours of treatment, to provide information about the relative efficacy of the machines with minimal opportunity for confounding. The secondary endpoints were: duration of phototherapy; change in TSB/hour over the whole duration of treatment; and LOS in the NCU. For each endpoint, a comparison of the three phototherapy machines and by a comparison of single vs. double sided machines were performed.

### Power

The power of the study was based on a planned recruitment of 300 infants. Because of the unbalanced allocation built into the design, it was noted that the Firefly would have the lowest number of infants. Means and standard deviations were unknown; allocation of 63 infants per group allowed detection of a difference of half a standard deviation in pairwise means; by enrolling 300 infants, we anticipated ≥ 63 infants in all three groups, and capacity to detect this difference or more.

### Statistical analysis

Categorical data were expressed as number and percentage, while continuous data as median and interquartile range (IQR). Demographics and risk factors for hyperbilirubinemia were compared among the three phototherapy groups using Fisher’s Exact test or Kruskal-Wallis test, as appropriate. Multivariable analysis of primary and secondary endpoints was performed using ordinary least squares (OLS) regression, adjusting for potential confounders that were reported in 10 or more infants. As the distributions were only approximately normal, statistical significance was confirmed using bootstrap methods to calculate 95% Confidence Intervals (CIs), which were compared to CIs produced by OLS. Sampling for bootstrap analysis was with replacement, undertaken within each allocation group to create 10,000 samples of the same size and treatment group composition as the original; the final OLS model was re-run on each of the samples and parameter estimates saved; for each of the parameters estimated, the mean of each parameter was calculated, and the 2.5th and 97.5th percentile were used as the bounds of the bootstrap 95% CIs [10]. The average change in endpoint was calculated using OLS parameter estimates and the mean value or prevalence for each potential confounder. The duration outcomes (duration of treatment and LOS in the NCU) were positively skewed, thus were log-transformed before analysis. Since the transformed outcomes did not meaningfully improve model fit or alter the statistical significance, results of untransformed models were reported to ease interpretation of results. Analysis was based on the principle of ‘intention to treat’ and included a comparison of the three phototherapy machines, followed by a comparison of single vs. double sided machines. All tests were two-tailed and analysis was performed using SAS 9.4 (SAS Institute, Cary, NC).

### Ethical approval

The Ethics Committee at the Da Nang Hospital for Women and Children approved the study and waived the need for consent given the observational design of the study.

## Results

### Patients

At study completion, 310 infants had been allocated to the three machines: 113 to the PTV3000, 104 to the Lullaby, and 93 to the Firefly. All infants were treated on the machines to which they were assigned; 26% commenced treatment <72 hours of age, and 25% commenced treatment >120 hours of age. Median TSB at the start of treatment was 287 μmol/L (IQR 255–328). Patient characteristics are shown in Table 2. Infants allocated to Firefly were less likely to commence treatment <72 hours of age (p<0.05) and had higher median TSB at start of treatment (p<0.001). No infant was: Rh(D) positive; born to a multiparous Rh(D) negative mother; having received oxygen; having a previous sibling who received phototherapy; having significant bruising; or breastfeeding exclusively and feeding poorly.

**Table 2:** Patient characteristics by treatment allocation.

		Total	Specific machine				Machine type		
			PTV3000	Lullaby	Firefly	p-value	Single	Double	p-value
n		310	113	104	93		217	93	
Place of birth	Inborn	247 (79.7)	92 (81.4)	83 (79.8)	72 (77.4)	0.70	175 (80.7)	72 (77.4)	0.42
	Outborn	57 (18.4)	19 (16.8)	18 (17.3)	20 (21.5)		37 (17.1)	20 (21.5)	
	Missing	6 (1.9)	2 (1.8)	3 (2.9)	1 (1.1)		5 (2.3)	1 (1.1)	
Sex	Male	183 (59.0)	59 (52.2)	63 (60.6)	61 (65.6)	0.14	122 (56.2)	61 (65.6)	0.17
	Female	126 (40.6)	54 (47.8)	40 (38.5)	32 (34.4)		94 (43.3)	32 (34.4)	
	Missing	1 (0.3)	0 (0.0)	1 (1.0)	0 (0.0)		1 (0.5)	0 (0.0)	
Birthweight,	Grams	3100	3170	3100	3109	0.44	3100	3109	0.60
		(2840–3400)	(2900–3400)	(2800–3355)	(2900–3400)		(2800–3400)	(2900–3400)	
35–38 weeks gestation	No	209 (67.4)	82 (72.6)	69 (66.3)	58 (62.4)	0.31	151 (69.6)	58 (62.4)	0.19
	Yes	99 (31.9)	31 (27.4)	33 (31.7)	35 (37.6)		64 (29.5)	35 (37.6)	
	Missing	2 (0.6)	0 (0.0)	2 (1.9)	0 (0.0)		2 (0.9)	0 (0.0)	
G6PD	Not tested	298 (96.1)	109 (96.5)	101 (97.1)	88 (94.6)	0.55	210 (96.8)	88 (94.6)	0.52
	Sufficiency	9 (2.9)	4 (3.5)	2 (1.9)	3 (3.2)		6 (2.7)	3 (3.2)	
	Deficiency	3 (1.0)	0 (0.0)	1 (1.0)	2 (2.2)		1 (0.5)	2 (2.2)	
ABO (Coombs +ve)	Not tested	9 (2.9)	3 (2.6)	6 (5.8)	0 (0.0)	0.75	9 (4.2)	0 (0.0)	0.51
	Negative	299 (96.5)	109 (96.5)	98 (94.2)	92 (98.9)		209 (96.3)	92 (98.9)	
	Positive	2 (0.6)	1 (0.9)	0 (0.0)	1 (1.1)		1 (0.5)	1 (1.1)	
Infection (treated with antibiotics)	No	272 (87.7)	95 (84.1)	93 (89.4)	84 (90.3)	0.33	188 (86.6)	84 (90.3)	0.45
	Yes	38 (12.3)	18 (15.9)	11 (10.6)	9 (9.7)		11 (13.4)	9 (9.7)	
TSB at start of treatment	μmol/L	287	277	276	308	<0.001	277	308	<0.001
		(255–328)	(255–327)	(250–301)	(275–346)		(253–317)	(275–346)	
Age at start of treatment	<72 hours	80 (25.8)	35 (31.0)	30 (28.9)	15 (16.1)	0.04	65 (29.9)	15 (16.1)	0.01
	72–120 hours	137 (44.2)	44 (38.9)	41 (39.4)	52 (55.9)		85 (39.2)	52 (55.9)	
	>120 hours	77 (24.8)	28 (24.8)	28 (26.9)	21 (22.6)		56 (25.8)	21 (22.6)	
	Missing	16 (5.2)	6 (5.3)	5 (4.8)	5 (5.4)		11 (5.1)	5 (5.4)	

Data expressed as n (%) or median (IQR). Comparisons were performed excluding missing data.

### Primary outcome

Over first six hours, Firefly lowered the TSB faster than PTV3000 (mean difference 1.51 μmol/L, 95% CI -2.56 to -0.45), while TSB decrease was similar with Lullaby and PTV3000 (mean difference 0.48 μmol/L, 95% CI -1.54 to 0.58) (Table 3). These findings were confirmed in double- vs. single-sided comparison (mean difference -1.29 μmol/L, 95% CI -2.34 to -0.26) (Table 3). OLS and bootstrap analyses showed comparable estimates.

**Table 3:** Change in TSB per hour (μmol/L) over first six hours.

		Univariate analysis	Multivariable analysis			
			3 machine comparison		Single vs double	
		OLS	OLS	Bootstrap	OLS	Bootstrap
		Pt. Est.	Pt. Est.	Pt. Est.	Pt. Est.	Pt. Est.
		(95% CI)	(95% CI)	(95% CI)	(95% CI)	(95% CI)
Intercept	Intercept	Various	-2.04 (-6.47 to 2.38)	-2.04 (-6.05 to 2.22)	-2.41 (-6.77 to 1.95)	-2.39 (-6.57 to 1.88)
Machine ^#^	PTV3000	Ref	Ref	Ref	-	-
	Lullaby	-0.18 (-1.34 to 0.97)	-0.50 (-1.55 to 0.55)	-0.48 (-1.54 to 0.58)		
	Firefly	-1.81 (-2.98 to -0.64)	-1.50 (-2.60 to -0.40)	-1.51 (-2.56 to -0.45)		
Machine type	Single-sided	Ref	-	-	Ref	Ref
	Double-sided	-1.72 (-2.76 to -0.69)			-1.27 (-2.26 to -0.28)	-1.29 (-2.34 to -0.26)
Place born	Inborn	Ref	Ref	Ref	Ref	Ref
	Outborn	-0.80 (-2.08 to 0.48)	-0.20 (-1.40 to 0.99)	-0.20 (-1.56 to 1.13)	-0.22 (-1.41 to 0.97)	-0.20 (-1.65 to 1.15)
Birthweight	kg	2.01 (0.79 to 3.23)	2.42 (1.27 to 3.57)	2.42 (1.09 to 3.75)	2.43 (1.28 to 3.58)	2.43 (1.03 to 3.77)
Sex	Female	Ref	Ref	Ref	Ref	Ref
	Male	0.39 (-0.59 to 1.38)	0.15 (-0.76 to 1.05)	0.15 (-0.77 to 1.07)	0.13 (-0.78 to 1.03)	0.13 (-0.80 to 1.06)
35–38 weeks	No	Ref	Ref	Ref	Ref	Ref
	Yes	0.74 (-0.29 to 1.77)	0.92 (-0.04 to 1.89)	0.92 (0.002 to 1.81)	0.92 (-0.05 to 1.88)	0.91 (-0.01 to 1.93)
Infection treated with antibiotics	No	Ref	Ref	Ref	Ref	Ref
	Yes	-1.55 (-3.00 to -0.11)	-2.04 (-3.42 to -0.66)	-2.03 (-3.58 to -0.55)	-1.99 (-3.36 to -0.62)	-1.98 (-3.48 to -0.54)
Age at start of phototherapy^	<72 hours	-0.30 (-1.46 to 0.85)	-0.26 (-0.88 to 1.39)	-0.25 (-0.80 to 1.32)	-0.26 (-0.88 to 1.39)	-0.25 (-0.83 to 1.32)
	72–120 hours	Ref	Ref	Ref	Ref	Ref
	>120 hours	-1.81 (-2.99 to -0.64)	-1.00 (-2.15 to 0.15)	-1.00 (-2.15 to -0.14)	-1.01 (-2.16 to 0.13)	-1.02 (-2.15 to 0.11)
TSB at start of phototherapy	/100 μmol/L	-2.81 (-3.66 to -1.96)	-2.56 (-3.43 to -1.69)	-2.56 (-3.78 to -1.35)	-2.52 (-3.39 to -1.66)	-2.52 (-3.70 to -1.35)

OLS: Ordinary Least Squares Regression. Pt. Est.: Point Estimate. 95% CI: 95% Confidence Interval.

^#^ Overall p-values for overall machine effect: univariate: p = 005; multivariable: p = 0.03.

^ Overall p-values for overall effect of age at start of treatment: univariate: p = 009; multivariable three-machine: p = 0.13; multivariable: p = 0.12.

### Secondary outcomes

As with the primary endpoint, the two single-sided machines (Lullaby than PTV3000) showed comparable results regarding the secondary endpoints (Tables 4–6). Double-sided phototherapy was associated with a 13.8-hour reduction in the duration of treatment (95% CI 5.7 to 22.8; Table 4). The average duration of treatment was 64.7 hours on single-sided phototherapy and 50.9 hours on double-sided, which represents a 21% reduction in treatment duration. More than half of all phototherapy treatments ceased on the hour, between 7–11 am, likely reflecting clinical schedules for patient review.

**Table 4:** Duration of phototherapy (hours).

		Univariate analysis	Multivariable analysis			
			3 machine comparison		Single vs double	
		OLS	OLS	Bootstrap	OLS	Bootstrap
		Pt. Est.	Pt. Est.	Pt. Est.	Pt. Est.	Pt. Est.
		(95% CI)	(95% CI)	(95% CI)	(95% CI)	(95% CI)
Intercept	Intercept	Various	3.0 (-35.2 to 41.2)	2.9 (-33.2 to 37.6)	2.2 (-35.4 to 39.7)	2.3 (-33.0 to 36.7)
Machine ^#^	PTV3000	Ref	Ref	Ref	-	-
	Lullaby	-4.5 (-14.1 to 5.1)	-1.1 (-10.1 to 7.9)	-1.0 (-10.7 to 8.2)		
	Firefly	-11.2 (-21.0 to -1.4)	-14.3 (-23.7 to -4.8)	-14.1 (-25.8 to -3.9)		
Machine type	Single-sided	Ref	-	-	Ref	Ref
	Double-sided	-9.1 (-17.8 to -0.4)			-13.8 (-22.3 to -5.3)	-13.8 (-22.8 to -5.7)
Place born	Inborn	Ref	Ref	Ref	Ref	Ref
	Outborn	-1.3 (-11.9 to 9.3)	-5.3 (-15.6 to 5.0)	-5.2 (-15.2 to 4.3)	-5.3 (-15.6 to 4.9)	-5.3 (-15.4 to 4.4)
Birthweight	kg	4.0 (-14.3 to 6.2)	-1.9 (-11.8 to 8.1)	-1.7 (-16.0 to 10.5)	-1.8 (-11.8 to 8.1)	-1.8 (-16.1 to 10.4)
Sex	Female	Ref	Ref	Ref	Ref	Ref
	Male	-6.0 (-14.1 to 2.1)	-3.2 (-11.0 to 4.6)	-3.3 (-10.3 to 4.3)	-3.2 (-11.0 to 4.6)	-3.3 (-10.4 to 4.2)
35–38 weeks	No	Ref	Ref	Ref	Ref	Ref
	Yes	4.9 (-3.7 to 13.4)	9.6 (1.2 to 17.9)	9.3 (0.4 to 19.3)	9.5 (1.2 to 17.8)	9.4 (0.6 to 19.4)
Infection treated with antibiotics	No	Ref	Ref	Ref	Ref	Ref
	Yes	20.0 (8.2 to 31.8)	20.4 (8.5 to 32.3)	20.5 (8.5 to 32.2)	20.5 (8.7 to 32.3)	20.5 (8.7 to 32.4)
Age at start of phototherapy^	<72 hours	9.6 (-0.1 to 19.2)	4.2 (-5.6 to 14.0)	4.1 (-4.0 to 12.6)	4.2 (-5.6 to 14.0)	4.1 (-4.0 to 12.8)
	72–120 hours	Ref	Ref	Ref	Ref	Ref
	>120 hours	7.6 (-2.2 to 17.4)	3.7 (-6.2 to 13.6)	3.5 (-5.7 to 14.0)	3.7 (-6.2 to 13.5)	3.7 (-5.7 to 14.1)
TSB at start of phototherapy	/100 μmol/L	17.1 (9.8 to 24.3)	21.4 (13.9 to 28.9)	21.2 (11.5 to 32.6)	21.5 (14.0 to 28.9)	21.4 (11.4 to 33.7)

OLS: Ordinary Least Squares Regression. Pt. Est.: Point Estimate. 95% CI: 95% Confidence Interval.

^#^ Overall p-values for overall machine effect: univariate: p = 0.08; multivariable: p = 0.007

^ Overall p-values for overall effect of age at start of treatment: univariate: p = 0.10; multivariable three-machine: p = 0.63; multivariable: p = 0.63.

**Table 5:** Change in TSB per hour (μmol/L) over treatment.

		Univariate analysis	Multivariable analysis			
			3 machine comparison		Single vs double	
		OLS	OLS	Bootstrap	OLS	Bootstrap
		Pt. Est.	Pt. Est.	Pt. Est.	Pt. Est.	Pt. Est.
		(95% CI)	(95% CI)	(95% CI)	(95% CI)	(95% CI)
Intercept	Intercept	Various	-0.48 (-2.07, 1.10)	-0.49 (-2.00, 1.00)	-0.65 (-2.22, 0.93)	-0.66 (-2.21, 0.83)
Machine ^#^	PTV3000	Ref	Ref	Ref	-	-
	Lullaby	-0.29 (-0.70, 0.11)	-0.28 (-0.66, 0.10)	-0.28 (-0.66, 0.07)		
	Firefly	-1.20 (-1.60, -0.79)	-1.08 (-1.48, -0.68)	-1.09 (-1.48, -0.73)		
Machine type	Single-sided	Ref	-	-	Ref	Ref
	Double-sided	-1.04 (-1.40, -0.70)			-0.94 (-1.29, -0.59)	-0.94 (-1.29, -0.62)
Place born	Inborn	Ref	Ref	Ref	Ref	Ref
	Outborn	-0.05 (-0.43, 0.52)	0.33 (-0.11, 0.76)	0.33 (-0.11, 0.76)	0.31 (-0.13, 0.75)	-0.31 (-0.12, 0.73)
Birthweight	kg	0.02 (-0.02, 0.4)	0.21 (-0.19, 0.61)	0.21 (-0.15, 0.60)	0.23 (-0.18, 0.63)	0.23 (-0.14, 0.62)
Sex	Female	Ref	Ref	Ref	Ref	Ref
	Male	-0.11 (-0.47, 0.26)	-0.09 (-0.42, 0.24)	-0.09 (-0.42, 0.24)	-0.11 (-0.44, 0.21)	-0.11 (-0.45, 0.23)
35–38 weeks	No	Ref	Ref	Ref	Ref	Ref
	Yes	0.27 (-011, 0.66)	0.34 (-0.02, 0.69)	0.34 (0.03, 0.65)	0.32 (-0.03, 0.68)	0.32 (0.02, 0.63)
Infection treated with antibiotics	No	Ref	Ref	Ref	Ref	Ref
	Yes	0.51 (-0.04, 1.07)	0.28 (-0.23, 0.80)	0.29 (-0.21, 0.79)	0.29 (-0.22, 0.80)	0.29 (-0.21, 0.79)
Age at start of phototherapy^	<72 hours	-0.16 (-0.26, 0.57)	-0.15 (-0.55, 0.24)	-0.16 (-0.60, 0.24)	-0.15 (-0.55, 0.24)	-0.15 (-0.61, 0.25)
	72–120 hours	Ref	Ref	Ref	Ref	Ref
	>120 hours	-0.58 (-1.02, -0.15)	-0.59 (-1.00, -0.17)	-0.59 (-1.02, -0.18)	-0.60 (-1.01, -0.19)	-0.60 (-1.04, -0.18)
TSB at start of phototherapy	/100 μmol/L	-1.00 (-1.32, -0.69)	-0.69 (-1.02, -0.37)	-0.69 (-1.05, -0.31)	0.69 (-1.02, -0.37)	-0.69 (-1.05, -0.29)

OLS: Ordinary Least Squares Regression. Pt. Est.: Point Estimate. 95% CI: 95% Confidence Interval.

^#^ Overall p-values for overall machine effect: univariate: p<0.0001; multivariable: p<0.0001

^ Overall p-values for overall effect of age at start of treatment: univariate: p = 0.006; multivariable three-machine: p = 0.02; multivariable: p = 0.02

**Table 6:** LOS in NCU (hours).

		Univariate analysis	Multivariable analysis			
			3 machine comparison		Single vs double	
		OLS	OLS	Bootstrap	OLS	Bootstrap
		Pt. Est.	Pt. Est.	Pt. Est.	Pt. Est.	Pt. Est.
		(95% CI)	(95% CI)	(95% CI)	(95% CI)	(95% CI)
Intercept	Intercept	Various	45.0 (-2.4, 92.4)	44.8 (-0.02, 90.3)	44.0 (-2.6, 90.7)	43.8 (0.8, 87.7)
Machine ^#^	PTV3000	Ref	Ref	Ref	-	-
	Lullaby	-7.9 (-21.4, 5.5)	-1.4 (-12.6, 9.9)	-1.5 (-13.6, 10.8)		
	Firefly	-15.2 (-28.9, -1.6)	-14.6 (-26.4, -2.8)	-14.5 (-27.2, -2.9)		
Machine type	Single-sided	Ref	-	-	Ref	Ref
	Double-sided	-11.5 (-23.7, 0.6)			-14.0 (-24.6, -3.4)	-13.7 (-24.7, -3.3)
Place born	Inborn	Ref	Ref	Ref	Ref	Ref
	Outborn	-0.2 (-15.0, 14.7)	-6.4 (-19.2, 6.4)	-6.5 (-20.7, 9.4)	-6.5 (-19.3, 6.3)	6.6 (-20.9, 9.4)
Birthweight	kg	-14.3 (-28.5, -0.02)	-10.8 (-23.1, 1.6)	-10.4 (-27.2, 4.5)	-10.7 (-23.0, 1.6)	-10.4 (-27.0, 4.7)
Sex	Female	Ref	Ref	Ref	Ref	Ref
	Male	-5.5 (-16.9, 5.9)	1.6 (-8.2, 11.3)	1.2 (-8.6, 12.1)	1.5 (-8.2, 11.2)	1.2 (-9.0, 11.9)
35–38 weeks	No	Ref	Ref	Ref	Ref	Ref
	Yes	0.5 (-12.5, 11.5)	3.5 (-6.9, 13.9)	3.4 (-7.4, 14.3)	3.5 (-6.9, 13.9)	3.4 (-7.4, 14.6)
Infection treated with antibiotics	No	Ref	Ref	Ref	Ref	Ref
	Yes	74.0 (60.4, 89.1)	76.2 (61.4, 91.0)	76.3 (55.7, 99.4)	76.3 (61.6, 91.0)	76.2 (55.9, 99.1)
Age at start of phototherapy^	<72 hours	21.5 (8.1, 34.9)	2.0 (-10.2, 14.2)	1.9 (-8.9, 13.2)	2.0 (-10.2, 14.2)	1.9 (-8.6, 12.9)
	72–120 hours	Ref	Ref	Ref	Ref	Ref
	>120 hours	9.3 (-4.2, 22.8)	5.9 (-6.4, 18.2)	5.8 (-7.0, 18.7)	5.9 (-6.4, 18.2)	5.8 (-7.0, 18.8)
TSB at start of phototherapy	/100 μmol/L	15.0 (46.1, 25.3)	21.6 (12.3, 30.9)	21.4 (8.3, 35.8)	21.7 (12.5, 31.0)	21.5 (8.3, 35.9)

OLS: Ordinary Least Squares Regression. Pt. Est.: Point Estimate. 95% CI: 95% Confidence Interval.

# Overall p-values for overall machine effect: univariate: p = 0.09; multivariable: p = 0.03

^ Overall p-values for overall effect of age at start of treatment: univariate: p = 0.007; multivariable three-machine: p = 0.64; multivariable: p = 0.64.

Double-sided phototherapy resulted in TSB reduction at a rate 0.94 μmol/L/hr faster (95% CI 0.62 to 1.29) than single-sided over the whole duration of treatment (Table 5). The average rate of TSB reduction was 2.1 μmol/L/hr on the single-sided phototherapy and 3.0 μmol/L/hr on the double-sided, representing a 45% increase in the rate of TSB reduction over the whole duration of treatment.

Double-sided phototherapy resulted in a 14-hour shorter LOS in the NCU (95% CI 3 to 25; Table 6). The average LOS in the NCU for this study population was 87 hours on single-sided phototherapy and 73 hours on double-sided, representing a 16% reduction in LOS. OLS and bootstrap analyses showed comparable estimates. The time of discharge from the NCU showed a likely practice schedule: 88% of discharges from the unit were at 7, 8 or 11am, or at 2 or 5 pm.

## Discussion

Neonatal jaundice is one of the most common conditions requiring hospital admission [1], and has been reported to be responsible for half of admissions to NCUs in some LRSs [8]. Prevention, accurate diagnosis and effective treatment are all important elements of jaundice management [11]. This study focused on effective treatment and compared different LED phototherapy machines designed for use in LRSs in term of speed of TSB reduction and duration of treatment. Speed of TSB reduction is important in preventing higher-risk interventions such as exchange transfusion, while duration of treatment and LOS impact workload in NCUs and the number of infants that can potentially benefit from treatment.

After adjusting for TSB at the start of treatment, and other potential confounders, the two single-sided machines (PTV3000 and Lullaby) showed no difference in the speed of TSB reduction, in duration of treatment and in LOS in the NCU.

Increasing the surface-area exposed to light has been routinely recommended for improving the effectiveness of phototherapy [12–14]. Several studies compared single- and double-sided phototherapy to increase surface area, with conflicting results. Some reported faster speed of reduction and/or shorter duration of treatment with double-sided treatment [15–20], but others showed no difference in a simple comparison [21]. or where the total irradiance was identical in the single or double-sided configurations [22], or where single-sided therapy was augmented with reflecting curtains [23].

Here we assessed, for first the first time, the effectiveness on TSB reduction of a single- vs double-sided LED phototherapy machines specifically designed for use in LRSs; robust and simple to use. After controlling for potential confounders, the double-sided machine had a rate of TSB reduction which was 54% faster in the first 6 hours of treatment, and 45% faster over the duration of treatment. After adjustment, double-sided treatment was also associated with an 14-hour reduction in the duration of treatment, and a 14-hour reduction in LOS in the NCU. It is notable that the PTV3000 has white curtains and a method of fixing them in place, and clinicians confirm that these curtains were routinely used during treatment. However, consistent differences were still found between PTV3000 and the double-sided Firefly in speed of TSB reduction, and in duration of treatment and LOS.

From a planning point of view, a combination of different types of phototherapy devices in an NCU is likely to be optimal, depending on the funds available and cost of the machines. An increasing number of different LED phototherapy machines are being developed for use in LRSs, but the cost and availability of any particular machine can vary enormously from one LRS to another. Where available and affordable, replacing conventional phototherapy with LED can solve the problem of ineffective phototherapy being provided in LRSs, reducing ongoing administrative and financing costs associated with the supply of replaceable bulbs. For infants that can thermoregulate, and in settings where ambient temperature is adequate, double-sided Firefly can lead to faster reduction in TSB, a shorter duration of treatment and a shorter LOS in the NCU.

Given the known and potential side-effects associated with phototherapy [24], clinical schedules which optimise treatment durations are justified. In the current study, all TSB testing was conducted in the hospital laboratory, with longer delays expected for TSB results requested at night, or on weekends. The availability of affordable and reliable TSB testing within the NCU, to determine whether it is appropriate to cease treatment, could help modify these schedules [25], In addition, we noted a strong preference for phototherapy to end at 7–11 am, implying that treatment may be continuing longer than required, to fit clinical schedules. Similarly, we identified a practice pattern for infants to be discharged at particular times of the day. If confirmed, more frequent clinical review could result in shorter treatment duration and shorter LOS. Moreover, given the overcrowding found in many NCUs in LRSs, and the associated exposure to nosocomial infection, more frequent NCU-discharge review of lower risk neonates could help decongest NCUs by ensuring that infants only stay as long as necessary. Once again, availability of affordable and reliable TSB testing within the NCU could facilitate this, by proving prompt assessments of TSB rebound.

Beyond the comparisons in this paper, it should be noted that other options are available for treatment of jaundice in LRSs, including promising results with UV-filtered sunlight to treat infant with TSB <257 μmol/L [26, 27]. For infants with higher TSB requiring rapid TSB reduction, it is common for clinicians to use multiple machine simultaneously, where available.

This study has some limitations. First, because the Firefly cannot be used with an overhead heater, or within an incubator, the results can only be generalised to environments that allow for treatment without personalised heating, and to infants that can thermoregulate within that environment. While exclusion criteria were set lower, the study only enrolled infants born at ≥35 weeks’ gestation, weighing ≥1,900g, so results can only be generalised to this population. To minimise workload, we did not collect data on infants who declined to participate or who were excluded, so we are unable to comment on the impact of refusals or exclusions. Second, an observational study design with un-blinded quasi-randomised allocation process was chosen for practical reasons. Average TSB and age at commencement of treatment resulted imbalanced across the treatment arms, but they were included as confounders in all multivariable analyses. Finally, the study did not constrain clinical decisions about the distance of the bulb units from the infant, or their use of high and low intensity modes (options available in the single-sided machines). Although these aspects may have affected some observed differences between single- and double-sided phototherapy, our findings nevertheless demonstrated the advantages of double-sided high-intensity phototherapy in actual use in a real world LRS.

## Conclusions

We found no difference in the effectiveness of the two single-sided LED machines in terms of speed of reduction of TSB, duration of treatment or LOS in the NCU. Compared to single-sided machines, the double-sided machine showed a 45–54% increase in the rate of TSB reduction and a 14-hour reduction in both the duration of treatment and LOS in the NCU. Long-lasting LED phototherapy units eliminate the need for frequent replacement of bulbs, making it an affordable technology for LRSs and creating the opportunity for high quality phototherapy to be consistently provided. To maximise the benefit in terms of appropriateness of care and reduction in average patient load in the NCU and treatment of more infants on a single device, introduction of more effective phototherapy should be accompanied by review of standard practices of patient review and discharge to optimise the timing of treatment cessation and patient discharge.

## Supporting information

S1 Checklist STROBE Research checklist. (DOCX)

S1 Dataset Dataset containing all data underlying the findings described in the manuscript. (XLS)

## Abbreviations

AAP: American Academy of Pediatrics
BOL: Breath of Life Program
CI: Confidence Interval
IQR: Interquartile range (1st quartile, 3rd quartile)
INGO: International Non-Governmental Organization
LOS: Length of stay
LRS: Low resource setting
NCU: Neonatal Care Unit
OLS: Ordinary Least Squares Regression
TSB: Total Serum Bilirubin

## References

[1] National Collaborating Centre for Women’s and Children’s Health. Neonatal Jaundice: NICE; 2010.

[2] Maisels MJ. Neonatal hyperbilirubinemia and kernicterus—not gone but sometimes forgotten. Early Human Development. 2009:85(11):727–732. 10.1016/j.earlhumdev.2009.09.003 19833460

[3] Bhutani VK. Kernicterus as a 'Never-Event': a newborn safety standard? Indian J Pediatr. 2005;72(1):53–56. 15684449 10.1007/BF02760581

[4] Olusanya BO, Ogunlesi TA, Kumar P, Boo N-Y, Iskander IF, de Almeida MF, et al Management of late-preterm and term infants with hyperbilirubinaemia in resource-constrained settings. BMC paediatrics. 2015;15(1):39. 10.1186/s12887-015-0358-z PMC4409776 25884679

[5] Slusher TM, Zipursky A, Bhutani VK. A global need for affordable neonatal jaundice technologies. Semin Perinatol. 2011;35(3):185–191. 10.1053/j.semperi.2011.02.014 21641493

[6] Owa JA, Adebami OJ, Fadero FF, Slusher TM. Irradiance readings of phototherapy equipment: Nigeria. Indian J Pediatr. 2011;78(8):996–998. 10.1007/s12098-011-0382-4 21340724

[7] Cline BK, Vreman HJ, Faber K, Lou H, Donaldson KM, Amuabunosi E, et al Phototherapy device effectiveness in Nigeria: irradiance assessment and potential for improvement. J Trop Pediatr. 2013;59(4):321–325. 10.1093/tropej/fmt027 23666953

[8] Arnolda G, Thein AA, Trevisanuto D, Aung N, Nwe HM, Thin AA, et al Evaluation of a simple intervention to reduce exchange transfusion rates among inborn and outborn neonates in Myanmar, comparing pre- and post-intervention rates. BMC Pediatrics. 2015;15(1):1–10. 26678312 10.1186/s12887-015-0530-5 PMC4683769

[9] Kumar P, Chawla D, Deorari A, Kumar P, Chawla D, Deorari A. Light-emitting diode phototherapy for unconjugated hyperbilirubinaemia in neonates. Cochrane Database of Systematic Reviews. 2011;12:CD007969. 10.1002/14651858.CD007969.pub2 PMC6885069 22161417

[10] Efron B, Tibshirani R. An introduction to the bootstrap, vol. 57 Oxon; 1994.

[11] Greco C, Arnolda G, Boo NY, Iskander IF, Okolo AA, Rohsiswatmo R, et al Neonatal Jaundice in Low- and Middle-Income Countries: Lessons and Future Directions from the 2015 Don Ostrow Trieste Yellow Retreat. Neonatology. 2016;110(3):172–180. 10.1159/000445708 27172942

[12] American Academy of Pediatrics Subcommittee on Hyperbilirubinemia. Management of hyperbilirubinemia in the newborn infant 35 or more weeks of gestation. Pediatrics. 2004;114(1):297–316. 15231951 10.1542/peds.114.1.297

[13] Maisels MJ, McDonagh AF. Phototherapy for neonatal jaundice. N Engl J Med. 2008;358(9):920–928. 10.1056/NEJMct0708376 18305267

[14] Bhutani VK. Phototherapy to prevent severe neonatal hyperbilirubinemia in the newborn infant 35 or more weeks of gestation. Pediatrics. 2011;128(4):e1046–1052. 10.1542/peds.2011-1494 21949150 PMC11412217

[15] Holtrop PC, Ruedisueli K, Maisels MJ. Double versus single phototherapy in low birth weight newborns. Pediatrics. 1992;90(5):674–677. 1408537

[16] Sarici SU, Alpay F, Unay B, Ozcan O, Gokcay E. Double versus single phototherapy in term newborns with significant hyperbilirubinemia. J Trop Pediatr. 2000;46(1):36–39. 10.1093/tropej/46.1.36 10730039

[17] Thaithumyanon P, Visutiratmanee C. Double phototherapy in jaundiced term infants with hemolysis. Journal of the Medical Association of Thailand. 2002;85(11):1176–1181. 12546314

[18] Boonyarittipong P, Kriangburapa W, Booranavanich K. Effectiveness of double-surface intensive phototherapy versus single-surface intensive phototherapy for neonatal hyperbilirubinemia. Journal of the Medical Association of Thailand. 2008;91(1):50–55. 18386544

[19] Nuntnarumit P, Naka C. Comparison of the effectiveness between the adapted-double phototherapy versus conventional-single phototherapy. Journal of the Medical Association of Thailand. 2002;85(Suppl 4):S1159–1166. 12549790

[20] Kang JH, Shankaran S. Double phototherapy with high irradiance compared with single phototherapy in neonates with hyperbilirubinemia. Am J Perinatol. 1995;12(3):178–180. 10.1055/s-2007-994446 7612090

[21] Silva I, Luco M, Tapia JL, Perez ME, Salinas JA, Flores J, et al Single vs. double phototherapy in the treatment of full-term newborns with nonhemolytic hyperbilirubinemia. J Pediatr (Rio J). 2009;85(5):455–458. 19830352 10.2223/JPED.1927

[22] Tan KL. Comparison of the effectiveness of single-direction and double-direction phototherapy for neonatal jaundice. Pediatrics. 1975;56(4):550–553. 1165959

[23] Abd Hamid IJ, MI MI, Ibrahim NR, Abd Majid N, Ramli N, Van Rostenberghe H. Randomised controlled trial of single phototherapy with reflecting curtains versus double phototherapy in term newborns with hyperbilirubinaemia. J Paediatr Child Health. 2013;49(5):375–379. 10.1111/jpc.12192 23573836

[24] Xiong T, Qu Y, Cambier S, Mu D. The side effects of phototherapy for neonatal jaundice: what do we know? What should we do? Eur J Pediatr. 2011;170(10):1247–1255. 10.1007/s00431-011-1454-1 21455834

[25] Coda Zabetta CD, Iskander IF, Greco C, Bellarosa C, Demarini S, Tiribelli C, et al Bilistick: a low-cost point-of-care system to measure total plasma bilirubin. Neonatology. 2013;103(3):177–181. 10.1159/000345425 23295342

[26] Slusher TM, Vreman HJ, Olusanya BO, Wong RJ, Brearley AM, Vaucher YE, et al Safety and Efficacy of Filtered Sunlight in Treatment of Jaundice in African Neonates. Pediatrics. 2014;133(6):1569–1574. 10.1542/peds.2013-3500 PMC4531268 24864170

[27] Slusher TM, Olusanya BO, Vreman HJ, Brearley AM, Vaucher YE, Lund TC, et al A Randomized Trial of Phototherapy with Filtered Sunlight in African Neonates. N Engl J Med. 2015;373(12):1115–1124. 10.1056/NEJMoa1501074 26376136

